# Motor grading of elbow flexion – is Medical Research Council grading good enough?

**DOI:** 10.1186/1749-7221-4-3

**Published:** 2009-05-13

**Authors:** Praveen Bhardwaj, Navin Bhardwaj

**Affiliations:** 1 Hand & Microsurgery, Ganga Hospital, Coimbatore, India; 2 Consultant Physiotherapist, Badli, New Delhi- 110042, India

## Abstract

Restoration of elbow flexion is top priority in reconstruction following brachial plexus injury. Medical Research Council (MRC) Grading is the most commonly used scale to grade muscle power. Though simple to use, it has several limitations. Each grade represents a very wide range and hence precludes accurate assessment of function and outcome following a given procedure. Wide range of Grade 4 is most worrisome. Definitely all grade 4 labeled can not equate to good functional results. With most of the nerve transfer procedures described now claiming grade 4 recoveries in more than 80% of the reported cases a need for more detailed and accurate assessment of this grade is greatly felt. A modified MRC grading system is described which is comprehensive and easy to use.

## 

Recovery of elbow flexion is considered as top priority in reconstruction following brachial plexus injury, hence lot of procedures have been described to restore it [1-4]. Nerve transfer is the most preferred method unless the patient presents very late. To assess the recovery of elbow flexion Medical Research Council Grading has been most commonly used worldwide. Serious limitations of MRC grading system have been expressed by many authors [5,6] but it continues to be in use because of its simplicity. Many modifications have been used by various authors [5-9] but none are widely used. We believe that for any grading system to be widely acceptable it need to be a modification of the existing MRC grading system as this has been fed into at least three generations of residents and all are very used to and comfortable using this scale, may be at cost of accuracy. In addition, the grading system has to be comprehensive, easy to use and reproducible.

We have been using a modified MRC grading scale to assess the recovery of elbow flexion following nerve transfer in our patients (Table 1). This is a very simple grading system which basically is an elaborated MRC scale. The grade 0 and 1 remains same. Division of Grade 2 & 3 is influenced by the active motion scale described by Curtis et al [9]. Grade 2 has been subdivided into three subdivisions; A, B & C based on the range of motion with gravity eliminated. Grade 3 has been similarly subdivided depending on the range of motion against gravity. The subdivision of Grade 4 is based on the patient's ability to lift the weight through full range of flexion on a biceps curl machine, with weights in 0.5 Kg increments, a commonly used machine in physiotherapy departments and gymnasiums to strengthen the biceps. Grade 4 has three subdivisions; A- if the patient is able to lift less than 30% weight of the normal side; B- if he is able to lift 30–60% weight of the normal side; and C- if he is able to lift more than 60% weight of the normal side. Grade 5 will mean normal strength i.e. able to lift the same amount of weight as the normal side.

**Table 1:** Modified Medical Research Council system of grading elbow flexion

Grade	Subdivision	Description
**0**	-	No contraction

**1**	-	Perceptible contraction in the muscle but no movement

**2**		**Gravity Eliminated**

	**A**	Motion less than or equal to half range
	**B**	Motion more than half range
	**C**	Full range of motion

**3**		**Against Gravity**
	**A**	Motion less than or equal to half range
	**B**	Motion more than half range
	**C**	Full range of motion

**4**		**Motion Against Resistance**
	**A**	Able to lift less than 30% weight of the normal side through full range
	**B**	Able to lift 30–60% weight of the normal side through full range
	**C**	Able to lift more than 60% weight of the normal side through full range

**5**		Normal strength

We have found this scale very easy to use and reproducible. It has several advantages; by subdividing grade 2 and 3 we are able to track the recovery better, this not only helps the treating team to assess the recovery but also gives lot of confidence to the patient by knowing that he is improving. This is an important part for any nerve injury management as the nerve recovery takes very long time, may be months before patient migrated from grade 2 to grade 3, in which period patient may be very anxious and doubtful. By further subdividing these two grades we can actually show the progressive recovery to the patient and boost his confidence. Also, it will allow comparing the rate of recovery following different nerve transfer techniques.

Grade 4 is the least defined of all the grades in MRC system because of its widespread range [5,6]. If a patient is able to lift 1 kg weight he is labeled as grade 4 and another patient who is able to lift 20 kg is also grade 4. The difference between these two is phenomenal, both from functional point of view and for assessment of the final outcome following a surgical procedure. The data of the experimental study conducted by MacAvoy and Green [5] showed that grade 4 alone represents 96% of the entire spectrum of potential strength of the particular muscle and hence demands subdivisions for more precise assessment and documentation. They suggested that gross subjective estimate of strength as percentage of the normal side would be more useful than the MRC scale. But we believe that it will be too subjective and preclude standard and reproducible assessment.

Subdividing the grade 4 into three subgroups based on the percentage of weight a person could lift on a biceps curl machine is definitely useful. It allows us to objectively assess and document the recovery and the final functional outcome. With most of the nerve transfer procedures described now claiming grade 4 recoveries in more than 80% of the reported cases [2-4,10-13] it is high time we get more detailed assessment of this grade lest we shall be comparing 'apples with oranges'. Definitely all grade 4 labeled can not equate to good functional results. This subdivision shall give us clearer picture of the functional recovery and dictate the supremacy of one procedure over the other. A grading system similar to this may be applied to other muscle assessment as well.

## Abbreviations

MRC Grade: Medical Research Council Grade

## Competing interests

The authors declare that they have no competing interests.

## Authors' contributions

PB: Conceived the idea, collected the relevant literature, designed the modified classification and wrote the article.

NB: Designed the modified classification and used it in the clinical practice.

Both the authors have read the final version of the article and agreed to its content.
